# Bildgebung in der prächirurgischen Epilepsiediagnostik

**DOI:** 10.1007/s00115-021-01180-3

**Published:** 2021-09-07

**Authors:** Maria Ilyas-Feldmann, Bernd Vorderwülbecke, Mirja Steinbrenner

**Affiliations:** 1 Epilepsie-Zentrum Berlin-Brandenburg, Klinik für Neurologie mit Experimenteller Neurologie, Charité – Universitätsmedizin Berlin, Charitéplatz 1, 10117 Berlin, Deutschland; 2 Unité d’EEG et d’épileptologie, Hôpitaux Universitaires et Faculté de Médecine, Université de Genève, Genf, Schweiz

**Keywords:** Epilepsie, Chirurgie, MRT, PET, EEG, Epilepsy, Surgery, MRI, PET, EEG

## Abstract

Während zwei Drittel der PatientInnen mit Epilepsie durch Medikamente anfallsfrei werden, ist die Erkrankung bei 30 % pharmakoresistent. Bei pharmakoresistenter fokaler Epilepsie bietet die Epilepsiechirurgie eine etwa 65 %ige Chance auf Anfallsfreiheit. Vorab muss der Anfallsfokus exakt eingegrenzt werden, wofür bildgebende Methoden unverzichtbar sind. In den letzten Jahren hat sich in der Prächirurgie der Anteil von PatientInnen mit unauffälliger konventioneller Magnetresonanztomographie (MRT) erhöht. Allerdings konnte die Sensitivität der MRT durch spezielle Aufnahmesequenzen und Techniken der Postprozessierung gesteigert werden. Die Quellenlokalisation des Signals von Elektro- und Magnetenzephalographie (EEG und MEG) verortet den Ursprung iktaler und interiktaler epileptischer Aktivität im Gehirn. Nuklearmedizinische Untersuchungen wie die interiktale Positronen-Emissions-Tomographie (PET) und die iktale Einzelphotonen-Emissionscomputertomographie (SPECT) detektieren chronische oder akute anfallsbezogene Veränderungen des Hirnmetabolismus und können auch bei nichtlokalisierendem MRT auf den epileptogenen Fokus hinweisen. Alle Befunde zusammengenommen werden zur Planung eventueller invasiver EEG-Ableitungen und letztlich der chirurgischen Operation eingesetzt. Konkordante Befunde sind mit besseren chirurgischen Ergebnissen assoziiert und zeigen auch im Langzeitverlauf signifikant höhere Anfallsfreiheitsraten.

## Abstract (en)

While two thirds of patients with epilepsy become seizure-free with antiseizure medications, 30% remain drug-resistant. In drug-resistant focal epilepsy, epilepsy surgery offers an approximately 65% chance of becoming seizure-free; however, for a successful outcome of surgery a seizure focus must be precisely located, for which imaging techniques are essential. In recent years, the proportion of patients with apparently inconspicuous findings in magnetic resonance imaging (MRI) in the presurgical evaluation has increased. The sensitivity of MRI can be increased using special MRI sequences and MRI postprocessing techniques. Ictal and interictal source localization based on electroencephalography (EEG) and magnetencephalography (MEG) aim at determining the onset of interictal discharges and seizures. Nuclear medicine imaging techniques such as interictal positron emission tomography (PET) and ictal single photon emission computed tomography (SPECT) can detect chronic or acute seizure-related changes in brain metabolism and can indicate an epileptogenic focus even if MRI is inconspicuous. The results of these techniques are used to plan invasive EEG recordings and subsequently surgery. Concordant findings are associated with better surgical outcomes and show significantly higher rates of seizure freedom in the long-term seizure outcome.

## Hintergrund

Epilepsie betrifft ca. 50 Mio. Menschen weltweit [9]. Etwa ein Drittel von ihnen hat trotz adäquater medikamentöser Therapie weiterhin Anfälle, die Epilepsie ist also pharmakoresistent [7]. Bei pharmakoresistenter fokaler Epilepsie stellt die chirurgische Entfernung oder Abtrennung bzw. die thermische Ablation des Anfallsfokus die erfolgversprechendste Therapieoption dar [32]. Im Vergleich zur Pharmakotherapie, mit der ab dem 3. eingesetzten Medikament jeder weitere Therapieversuch nur mit 2–4 % Wahrscheinlichkeit Anfallsfreiheit bewirkt, sind 1 Jahr nach einer Operation im Mittel ca. 64 % der PatientInnen anfallsfrei (13–92 %, je nach Konstellation; [7, 32]). Jedem Eingriff geht eine gründliche multimodale Diagnostik mit Langzeit-Video-Elektroenzephalogramm (EEG), Magnetresonanztomographie (MRT) und neuropsychologischer Testung voraus. Einer der relevantesten Prädiktoren für eine erfolgreiche Epilepsiechirurgie ist die vollständige Entfernung einer vorher im MRT eindeutig identifizierten epileptogenen Hirnläsion [32]. Allerdings ist in den letzten Jahren der Anteil prächirurgischer PatientInnen mit klarer MRT-bildgebender Läsion seltener geworden [8]. In „MRT-negativen“ Fällen helfen verfeinerte MRT-Verfahren, die Kombination von Bildgebung mit elektrophysiologischen Methoden sowie nuklearmedizinische Messungen, die epileptogene Zone einzugrenzen und ggf. eine gezielte invasive EEG-Ableitung mit intrakraniellen Elektroden zu planen.

Im Folgenden geben wir einen Überblick über den aktuellen Stand der Technik und die neuesten Entwicklungen der zerebralen Bildgebung in der prächirurgischen Diagnostik, welche sich in Zielsetzung und technischen Ansprüchen von der Bildgebung z. B. nach erstem epileptischem Anfall unterscheidet.

## Strukturelle und funktionelle MRT

Die Kernspin- oder Magnetresonanztomographie (MRT) basiert auf dem unterschiedlichen Verhalten verschiedener Gewebetypen in einem starken Magnetfeld. Während die strukturelle MRT zur zwei- oder dreidimensionalen Abbildung anatomischer Strukturen dient, kann die funktionelle MRT (fMRT) die Sauerstoffsättigung des Blutes mit einer zeitlichen Auflösung im Sekundenbereich nachvollziehen und damit Muster der Hirnaktivierung darstellen.

### Strukturelle MRT

Angaben zum Vorhandensein epileptogener Läsionen in der strukturellen MRT bei bekannter Epilepsie variieren deutlich (17–91 %; [10, 30]). Systematische Untersuchungen konnten zeigen, dass sich diese hohe Variabilität zu großen Teilen durch das jeweils angewandte MRT-Protokoll (Standard-MRT vs. differenziertes Epilepsieprotokoll) und die Erfahrung der BefunderInnen erklären lässt [30]. Um die MRT-Bildgebung bei Epilepsie über Geräte und Länder hinweg zu vereinheitlichen und die diagnostische Ausbeute zu verbessern, empfahl die Neuroimaging Task Force der International League Against Epilepsy (ILAE) 2019 die Einführung des sog. „HARNESS“-Protokolls [3]. Eine vergleichende Gegenüberstellung mit dem in der aktuellen S1-Leitlinie der Deutschen Gesellschaft für Neurologie (DGN) von 2017 empfohlenen Protokoll [13] zeigt Tab. 1. Da das MRT nach HARNESS mehrheitlich als 3‑D-Datensatz aufgezeichnet wird, können die Bilder post hoc ohne Informations- oder Qualitätsverlust in jeder Ebene dargestellt werden; folglich ist bei der Aufnahme auch keine spezielle Angulierung (Winkelung) notwendig. Die geringe Schichtdicke von höchstens 1 mm führt zu einer Reduktion von Artefakten.

**Table 1:** 

„HARNESS“ – Harmonized Neuroimaging of Epilepsy Structural Sequences 2019 [3]	MRT-Protokoll bei Epilepsie nach DGN-Leitlinie 2017 [13]
Keine spezielle Angulierung notwendig	Teils mit temporaler Angulierung
Hochauflösende 3‑D-Sequenzen mit isotropen Voxeln (1 × 1 × 1 mm)	Höchstens 2‑mm-Schichtdicke
3‑D-T1	T1 sagittal
–	T1 koronar
3‑D-FLAIR	FLAIR axial
–	FLAIR koronar
T2/STIR koronar	T2-TSE axial
–	T2-TSE koronar (temporale Angulierung)
Optionale Sequenzen bei speziellen Fragestellungen: T2*/SWI oder T1 KM	–

**Figure 1:**
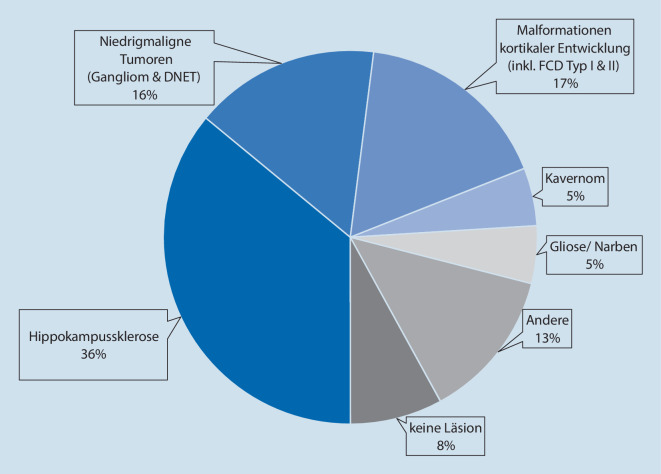

**Figure 2:**
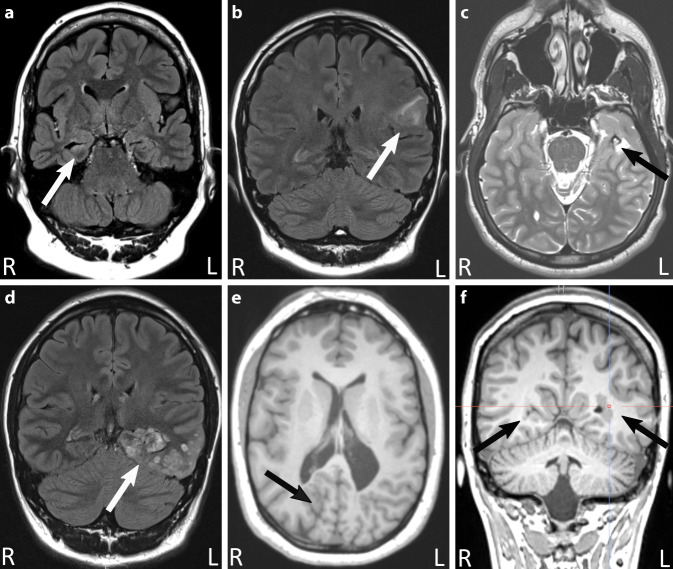

Die häufigste epileptogene Entität in der Epilepsiechirurgie ist die Hippokampussklerose, dicht gefolgt von niedrigmalignen Tumoren und Malformationen der kortikalen Entwicklung (Abb. 1, [4]). Radiologisch hinweisend auf epileptogene Läsionen in der strukturellen MRT sind abnormale Signalhyper- oder -hypointensitäten, subkortikale abnormale graue Substanz, kortikale Verdickungen, Störung der Grau-Weiß-Differenzierung, abnormale Gyrierung und lobäre Atrophien. Beispiele epileptogener Läsionen, die mit diesen Charakteristika im MRT einhergehen, sind in Abb. 2 dargestellt.

Trotz adäquatem Protokoll und Befundung durch ExpertInnen wird in bis zu 50 % der Fälle im Rahmen der prächirurgischen Diagnostik keine Läsion in der MRT gefunden [2]. Hier können Methoden der Postprozessierung die Sensitivität der MRT erhöhen. Eingang in die prächirurgische Diagnostik haben vor allem die Hippokampusvolumetrie [34] und das „morphometric parametric mapping“ (MAP) [19] gefunden. Die Volumenbestimmung des Hippokampus hilft, subtile bzw. beginnende Hippokampusatrophien nicht zu übersehen. Spätestens seit ein halbautomatisierter Ansatz die zeitaufwendige schichtweise manuelle Markierung ersetzt hat, ist sie unkompliziert anwendbar [34]. Die Morphometrie wiederum stellt Unterschiede in der Differenzierung von grauer und weißer Substanz dar. Dies dient insbesondere der Detektion fokaler kortikaler Dysplasien. Die durch MAP identifizierten Bereiche werden erneut visuell begutachtet, was in bis zu 24 % der vorher als unauffällig befundeten MRTs zur nachträglichen Detektion von Läsionen führt [19]. Weitere computergestützte Methoden insbesondere aus dem Bereich künstlicher Intelligenz („machine learning“, „deep learning“) werden zunehmend im Rahmen wissenschaftlicher Projekte untersucht, um hierüber die Läsionsdetektion zu verbessern [20].

Als weitere Unterform der strukturellen MRT können diffusionsgewichtete Aufnahmen Fasertrakte der weißen Substanz (z. B. der Sehbahn oder der Pyramidenbahn) darstellen, um sie bei der Operation möglichst zu verschonen und das Risiko postoperativer Defizite gering zu halten [11]. Eine Neuentwicklung im Bereich der strukturellen MRT ist die Hochfeldtomographie mit 7‑T-magnetischer Flussdichte, die eine bis zu 65 % höhere Detektionsrate epileptogener Läsionen erlaubt, bisher aber nur begrenzt Eingang in den klinischen Alltag gefunden hat [25].

### Funktionelle MRT

Die fMRT wird in der prächirurgischen Diagnostik häufig zur Identifizierung der sprachdominanten Hemisphäre und relevanter motorischer Areale (z. B. Handareal) angewandt [35]. In simultaner Aufzeichnung mit dem EEG kann die fMRT auch zur Lokalisation der irritativen Zone und bis zu einem gewissen Grad auch der Anfallsursprungszone eingesetzt werden (siehe Glossar). Hierbei macht man sich die hohe räumliche Auflösung der fMRT und die hohe zeitliche Auflösung des EEG zunutze. Epileptische Entladungen im EEG werden mit den gleichzeitig im fMRT messbaren regionalen Veränderungen der Blutoxygenierung in Zusammenhang gestellt [24]. Angaben zur Sensitivität der Methodik zur Fokuslokalisation schwanken von 40–90 % [35].

## EEG- und MEG-Quellenlokalisation

Die Ursprungslokalisation von Signalen des EEG oder Magnetenzephalogramms (MEG) ist strenggenommen keine bildgebende, sondern eine neurophysiologische Methode: Computerbasierte Algorithmen lokalisieren in einem 3‑D-Modell des Gehirns die Quellen des EEG- oder MEG-Signals in Form elektromagnetischer Dipole [18].

### EEG-Quellenlokalisation

**Figure 3:**
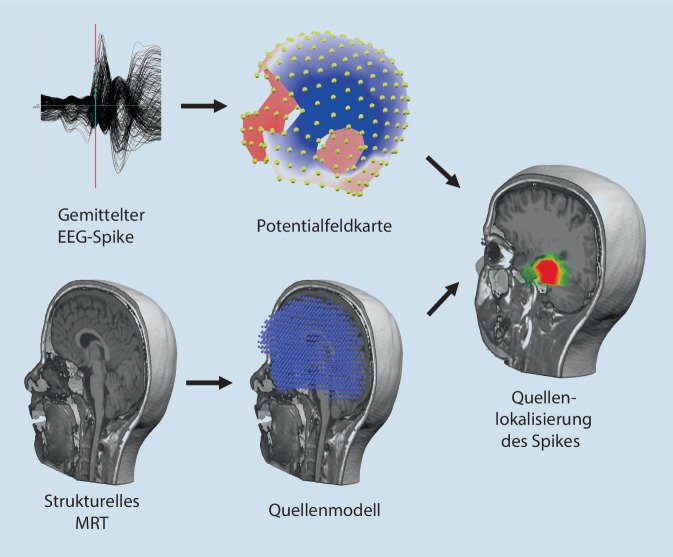

Das iktale Langzeit-Video-EEG gehört zum Kern der prächirurgischen Epilepsiediagnostik: Anfallssemiologie und EEG-Anfallsmuster erlauben Rückschlüsse auf die Anfallsursprungzone. Auch wenn das EEG-Signal in beliebigen Montagen dargestellt werden kann (z. B. unipolar, bipolar), ist die lokalisatorische Aussagekraft der klassischen visuellen EEG-Analyse begrenzt. Als Hilfsmittel kann die räumliche Verteilung der Potenziale im Oberflächen-EEG zu jedem beliebigen Zeitpunkt als Potenzialfeldkarte dargestellt werden (Abb. 3). Je nach Ort, Ausrichtung und Stärke der zerebralen Dipole ergeben sich charakteristische Muster.

Ausgehend von der Potenzialfeldkarte berechnet die EEG-Quellenlokalisation „rückwärts“, welche Potenzialgeneratoren (= Dipole) im Gehirn das EEG-Signal zum untersuchten Zeitpunkt am besten erklären. Hierzu stehen verschiedene biophysikalische Modelle und Algorithmen zur Verfügung, z. B. solche mit einzelnen Dipolen und solche mit tausenden [18]. Die Anatomie des Kopfes wird möglichst präzise modelliert, um die Volumenkonduktion der Potenziale durch die verschiedenen Kompartimente (Hirngewebe, Liquor, Knochen, Skalp etc.) nachzuvollziehen. Die Genauigkeit der EEG-Quellenlokalisation steigt, wenn ihr das individuelle strukturelle MRT der untersuchten Person zugrunde liegt [5].

Für die EEG-Quellenlokalisation werden zumeist interiktale Entladungen (Spikes oder scharfe Wellen) herangezogen, weil sie häufig auftreten, simpel konfiguriert sind und leicht gemittelt werden können [23]. Allerdings stimmt die Ursprungszone von Spikes und scharfen Wellen („irritative Zone“) nicht zwangsläufig mit der Ursprungszone der Anfälle überein. Laut einer großen Metaanalyse liegt die Sensitivität der interiktalen EEG-Quellenlokalisation, definiert als Anteil der Fälle mit Übereinstimmung von Lokalisierung und Resektionsgebiet unter allen postoperativ anfallsfreien PatientInnen, bei 81 % [28]. Auch iktale EEG-Quellenlokalisation ist möglich, aber im Vergleich zur interiktalen deutlich anspruchsvoller. EEG-Anfallsmuster sind heterogen konfiguriert und oft von Artefakten überlagert; den optimalen Zeitabschnitt festzulegen ist nicht trivial. Verschiedene mathematische Zwischenschritte zwischen EEG-Aufzeichnung und Quellenlokalisation werden vorgeschlagen, um das eigentliche iktale Signal von anderen zerebralen und extrazerebralen Signalkomponenten zu unterscheiden [29]. Während die iktale EEG-Quellenlokalisation derzeit noch Expertenkenntnisse voraussetzt, ist die interiktale Quellenlokalisation mittels einer kommerziellen oder nichtkommerziellen Software relativ einfach handhabbar. In einer prospektiven Untersuchung brachte die EEG-Quellenlokalisation bei einem Drittel der untersuchten prächirurgischen PatientInnen einen diagnostischen Zusatznutzen [14].

### MEG-Quellenlokalisation

Im Unterschied zum EEG zeichnet das MEG nicht Potenzialdifferenzen auf, sondern Magnetfelder. Weil elektrisches Feld und Magnetfeld eines Dipols im rechten Winkel zueinanderstehen, kann das MEG Signalquellen identifizieren, die dem EEG verborgen bleiben und umgekehrt [26]. Mathematisch folgen EEG- und MEG-Quellenlokalisation sehr ähnlichen Prinzipien. Weil im MEG-Scanner nur selten verwertbare EEG-Anfallsmuster aufgezeichnet werden können, basiert die MEG-Quellenlokalisation fast ausschließlich auf interiktalen Entladungen. Die Sensitivität liegt bei 65–77 % und ist höher bei extratemporalen als bei temporalen Epilepsien [27, 28]. Insgesamt begrenzt die limitierte Verfügbarkeit der teuren Scanner die MEG-Diagnostik.

## PET und SPECT

Nuklearmedizinische Untersuchungsmethoden erlauben, funktionell den Metabolismus von Geweben darzustellen, indem sich radioaktiv markierte Tracer in Kompartimenten oder Organregionen anreichern. Der Zerfall der Isotope wird mit Detektoren gemessen, was eine örtliche Darstellung der Aktivitätsverteilung ermöglicht. Die Positronen-Emissions-Tomographie (PET) und die Einzelphotonen-Emissionscomputertomographie (SPECT) sind vor allem bei MRT-negativen Epilepsien hilfreiche ergänzende Methoden, epileptogene Läsionen zu lokalisieren.

### Fluorodeoxyglucose-PET

Schon vor der Erfindung der MRT wurde in der prächirurgischen Epilepsiediagnostik die ^18^Fluoro-Deoxyglucose-PET (FDG-PET) verwendet. Bis heute ist FDG der am häufigsten eingesetzte Radioligand: Am Glukosemolekül ist eine Hydroxygruppe durch radioaktives 18-Fluor ersetzt [12]. Intravenös verabreicht, reichert es sich in glukoseverbrauchenden Geweben an. Der charakteristische Befund bei fokalen Epilepsien ist eine umschriebene Verringerung der interiktalen Glukoseaufnahme (Hypometabolismus). Die Sensitivität von PET zur Detektion epileptogener Hirnareale liegt zwischen 60–90 % bei Menschen mit Temporallappenepilepsie [15]. Die mittels FDG-PET dargestellten hypometabolen Areale sind meistens deutlich ausgedehnter als korrespondierende strukturelle Läsionen in der MRT [6]. Bei eindeutiger MRT-Läsion und konkordantem Video-EEG-Befund ist eine FDG-PET nicht notwendig, hingegen kann sie in MRT-negativen Fällen sehr hilfreich sein: Deuten sowohl elektroklinische Befunde als auch PET-Hypometabolismus auf eine Temporallappenepilepsie hin, ist die postoperative Anfallsprognose genauso gut wie bei PatientInnen mit MRT-darstellbarer Hippokampussklerose [33]. Da die zerebrale Aufnahme des Radioliganden über mehrere Minuten nach der Injektion hinweg erfolgt, erbringt ein iktales FDG-PET gegenüber einem interiktalen in der Regel keine zusätzlichen Informationen.

### PET mit anderen Radioliganden

In den vergangenen Jahren wurden verschiedene neuartige PET-Rezeptor-Liganden für GABAerge, serotonerge und andere Neurotransmittersysteme entwickelt, von denen aber bisher nur wenige in der klinischen Routine eingesetzt werden. ^11^C‑Flumazenil (FMZ) bindet an den zentralen Benzodiazepinrezeptor, einen Teil des GABA-A-Rezeptor-Komplexes. Die FMZ-Bindung ist bei der Hippokampussklerose und vaskulären Läsionen verringert, kann aber in Arealen kortikaler Dysgenesien erhöht sein [21]. Bei kortikalen Malformationen und Entwicklungsstörungen sind die metabolischen Veränderungen deutlicher ausgeprägt als die im MRT zum Teil nur schwer ersichtlichen strukturellen Auffälligkeiten [17]. Alpha-Methyl-L-Tryptophan (AMT), ein Analogon der essenziellen Aminosäure Tryptophan, ist die Vorstufe von Serotonin. Bei PatientInnen mit multifokaler Epilepsie, wie z. B. bei tuberöser Sklerose, kann die erhöhte Aufnahme von AMT im PET den aktivsten Fokus anzeigen [1]. Bei Kindern mit unifokaler Epilepsie und unauffälligem MRT-Befund zeigte ein Viertel eine fokal erhöhte AMT-Bindung, die spezifisch mit dem epileptogenen Fokus assoziiert war [31].

### SPECT

Die SPECT-Bildgebung kann mithilfe der Perfusionstracer ^99m^ Tc-Hexamethyl-Propylenamin-Oxim [HMPAO] oder ^99m^ Tc-Ethylen-Cystein-Diethylester [ECD] den regionalen zerebralen Blutfluss darstellen. Dieser ist während epileptischer Anfälle im übererregten Areal erhöht, und die mittels SPECT dargestellte iktale Hyperperfusion hat eine hohe lokalisatorische Aussagekraft [16]. Um die Anfallsursprungzone darzustellen, muss der Tracer möglichst innerhalb weniger Sekunden nach Anfallsbeginn injiziert werden. Anschließend kann das Auslesen im Scanner innerhalb von 2 h erfolgen. Da der regionale zerebrale Blutfluss bei Epilepsie auch interiktal verändert sein kann, muss zum Vergleich mit den iktalen Bildern eine interiktale SPECT nach einem mindestens 24-stündigen anfallsfreien Intervall angefertigt werden. Eine computergestützte Nachverarbeitung unter Zuhilfenahme des MRT, „subtraction ictal SPECT coregistered with MRI“ (SISCOM), erhöht die diagnostische Aussagekraft signifikant [22]. Eine Metaanalyse zeigte für SPECT eine Sensitivität von 44 % (interiktal), 75 % (postiktal) und 97 % (iktal) bei Menschen mit Temporallappenepilepsie. Wegen des hohen Aufwands wird das iktale SPECT in der prächirurgischen Epilepsiediagnostik vor allem bei MRT-negativen Fällen bzw. diskordanten Befunden eingesetzt [12].

## Fazit für die Praxis

In der prächirurgischen Epilepsiediagnostik helfen spezielle Epilepsieprotokolle und Methoden der Postprozessierung, die diagnostische Ausbeute der strukturellen MRT zu erhöhen. Ist weiterhin keine Läsion zu finden oder ergeben sich widersprüchliche Befunde zu Video-EEG und Neuropsychologie, werden andere nichtinvasive Methoden zur Fokuslokalisierung hinzugezogen. Dies sind elektrophysiologische Verfahren wie die EEG-Quellenlokalisation und nuklearmedizinische Methoden wie interiktale PET und iktale SPECT. Besteht weiterhin keine Klarheit, wird eine invasive EEG-Diagnostik mit implantierten Elektroden nötig. Die nichtinvasiven bildgebenden Verfahren werden in Zukunft weiterentwickelt und verfeinert werden, um möglichst vielen PatientInnen eine invasive EEG-Diagnostik ersparen zu können.

## Glossar

Anfallsursprungszone: Gehirnareal, in dem epileptische Anfälle beginnen (eingrenzbar durch EEG, EQL, SPECT sowie potenziell MEG und fMRT; jeweils iktal).
Epileptogene Läsion: Strukturelle Anomalie, welche Anfälle verursacht (eingrenzbar durch strukturelle MRT).
Epileptogene Zone („Anfallsfokus“): [25] Areal, dessen Entfernung notwendig und ausreichend ist, um anhaltende Anfallsfreiheit zu erreichen. Die epileptogene Zone ist ein hypothetisches Konstrukt. Anfallsursprungszone, irritative Zone, (potenziell) epileptogene MRT-Läsion, PET-Hypometabolismus etc. dienen als Surrogate.
Irritative Zone: Gehirnareal, in welchem interiktale epileptische Potenziale entstehen (eingrenzbar durch EEG, EQL, MEG, fMRT; jeweils interiktal).
